# Reply to: Concerns about the Burden of Proof studies

**DOI:** 10.1038/s41591-023-02295-7

**Published:** 2023-04-14

**Authors:** Aleksandr Y. Aravkin, Susan A. McLaughlin, Peng Zheng, Haley Lescinsky, Michael Brauer, Simon I. Hay, Christopher J. L. Murray

**Affiliations:** 1 Institute for Health Metrics and Evaluation, University of Washington, Seattle, WA USA; 2 Department of Health Metrics Sciences, School of Medicine, University of Washington, Seattle, WA USA; 3 Department of Applied Mathematics, University of Washington, Seattle, WA USA; 4 The University of British Columbia, Vancouver, British Columbia Canada

**Keywords:** Risk factors, Diseases

replying to A. J. Glenn et al. *Nature Medicine* 10.1038/s41591-023-02294-8 (2023)

We, together with the Global Burden of Disease Study and Burden of Proof (BoP) collaborators, have published a BoP Capstone Methods paper and suite of associated meta-analyses that introduce and apply a new framework for synthesizing evidence to evaluate relationships between selected risk factors and health outcomes^1–5^. The BoP approach was designed to address key problems in current analytical frameworks. To provide helpful information to users making decisions around risk exposure, the approach systematically estimates a flexible mean risk–outcome function, avoiding making strong assumptions such as log-linearity of the relationship. Complementing the mean risk–outcome relationship, BoP analysis provides a BoP risk function (BoPRF) that represents the lowest estimate of excess harmful risk associated with a risk factor, incorporating unexplained between-study heterogeneity after accounting for known variation in study design characteristics. From the BoPRF, we calculate summary risk–outcome scores and star-rating measures that present conservative estimates of the risk–outcome relationship and enable comparisons across different risk–outcome pairs.

In their Matters Arising on the BoP papers, Glenn et al.^6^ argue that our inclusion of between-study heterogeneity improperly inflates uncertainty intervals and that our star-rating summary measure is too simplistic. They further suggest that we wrongly assume risk–outcome relationships to be non-log-linear. We welcome this opportunity to engage in discourse and clarify misunderstandings. We all agree on the importance of using methods that summarize the available risk–outcome evidence in a way that most accurately captures the health risks people experience because of risk factor exposures.

With respect to the critique by Glenn et al. that our method improperly inflates uncertainty intervals, we note that in classic meta-analysis methods, between-study heterogeneity affects posterior uncertainty only marginally by enlarging the reported standard errors. As studies accumulate in the literature, even if each study contradicts the previous one, classic posterior uncertainty around effect size estimates continues to shrink, making the basis for health risk guidance ever more certain despite apparent disagreement between studies. The phenomena of strong recommendations in spite of highly variable conclusions of emerging studies lead, justifiably, to broad critiques of the entire field^7^. The status quo in meta-analytic research—where the inclusion of more studies creates increased precision in the estimate even if they are divergent—is counterintuitive. Classic confidence intervals by themselves often fail to capture what matters to scientists and the public because they largely ignore variation in estimates of effects between studies. We believe the BoP approach addresses this problem by providing a complementary summary measure that incorporates between-study heterogeneity to obtain a prediction interval at the study level^1^. This interval answers the question: ‘If a large criterion standard study was conducted today, how varied might its findings be, based on all available information?’ In our reports^1–5^ and visualizations (https://vizhub.healthdata.org/burden-of-proof/), we present both classically reported uncertainty intervals and the BoP uncertainty interval. The BoP approach provides new insight, allowing users to differentiate between research fields where results are consistent from those where they are not.

Specific study results may vary in part due to study design, length of follow-up and other topic-specific observable variables that correlate exactly to categories that Grading of Recommendations, Assessment, Development, and Evaluations^8,9^ and the Cochrane collaboration^10^ currently use to evaluate study quality. The BoP approach provides a mechanism to account for differences between study-level characteristics, to the extent that such differences are known and can be encoded to explain between-study heterogeneity. Any remaining unexplained between-study heterogeneity then contributes to the overall rating of effect size and evidence strength. Unexplained variation in results that remains after accounting for study-level covariates can (and should) contribute to a lower risk score and lower star ratings. We believe that users of the results will appreciate this transparency.

Glenn et al. incorrectly conclude that we assume that relationships between red meat consumption and chronic disease are not log-linear. A central principle in the BoP approach is to forgo the assumption that risks are log-linear; however, this is very different from assuming risks are ‘not log-linear’. As simulation studies in the Capstone paper^1^ clearly show, when the dose–response relationship is log-linear, we recover that shape. A major contribution of the BoP method is that it allows more nuanced assessment of the underlying risk, letting the data inform the shape of the relationship rather than the analyst imposing it. Applying the BoP approach to analyze the risk–outcome pairs included in the Global Burden of Disease Study, we see that most relationships are not in fact log-linear, but some are, including systolic blood pressure versus heart disease (at least, for systolic blood pressure between 120 and 170 mmHg)^4^. We suggest that indiscriminately assuming log-linearity is a generic problem in many canonical systematic reviews^11–15^, which may assume a log-linear relationship for computational convenience. Shortcomings of previous methodological attempts to account for non-log-linear relationships are discussed in the methods appendix of the Capstone paper^1^ and illustrated using simulation.

Because of the inherent limitations around correcting for study-level bias in meta-analyses and, in terms of dietary risk analyses, including studies that do not carefully consider alternative foods or conduct substitution analyses, Glenn et al. suggest that a better approach would be to obtain primary, individual-level data for all original studies and use standard methods to define variables and adjust for covariates. Pooled studies can be helpful for exploring bias and answering myriad nuanced questions that can be addressed with primary detailed data shared among investigators. However, depending on how pooled studies are conducted, the results may also mask a large amount of heterogeneity in findings across studies that deserves explanation. We share Glenn et al.’s concern about the underlying literature and note it as a limitation^1^. The critique stated here applies to studies that do not consider diet substitution (that is, most available studies) and to any meta-analyses that use such studies. Unfortunately, detailed individual-level information is rarely available, although ongoing efforts to synchronize and collaborate between investigators are commendable and promising. Nonetheless, most work in meta-analysis relies on extracting information across studies where only results were reported and individual-level data are unavailable.

We respect that these investigators feel strongly about the health risks of red meat consumption and recognize that they are looking for an explanation as to why the results of the BoP analysis do not agree with their previous beliefs. Hypotheses stand only until they are refuted; therein lies the dynamic of scientific debate and progress. There are certainly groups that feel differently from Glenn et al.^16,17^, not to mention the disagreements surrounding myriad other risk–outcome pairs^11–17^. The BoP approach incorporates divergent study results and study designs, accounts for explainable differences and allows comparison between strength of evidence across different scientific domains.
